# Model-based extension of high-throughput to high-content data

**DOI:** 10.1186/1752-0509-4-106

**Published:** 2010-08-05

**Authors:** Andrea C Pfeifer, Daniel Kaschek, Julie Bachmann, Ursula Klingmüller, Jens Timmer

**Affiliations:** 1 Division Systems Biology of Signal Transduction, DKFZ-ZMBH Alliance, German Cancer Research Center, Im Neuenheimer Feld 280, 69120 Heidelberg, Germany; 2 Bioquant, Heidelberg University, BioQuant Building, Im Neuenheimer Feld 267, 69120 Heidelberg, Germany; 3 Physics Institute, University of Freiburg, Hermann-Herder-Strasse 3, 79104 Freiburg i.Br., Germany; 4 Freiburg Institute for Advanced Studies (FRIAS), University of Freiburg, Albertstrasse 19, 79104 Freiburg i.Br., Germany

## Abstract

**Background:**

High-quality quantitative data is a major limitation in systems biology. The experimental data used in systems biology can be assigned to one of the following categories: assays yielding average data of a cell population, high-content single cell measurements and high-throughput techniques generating single cell data for large cell populations. For modeling purposes, a combination of data from different categories is highly desirable in order to increase the number of observable species and processes and thereby maximize the identifiability of parameters.

**Results:**

In this article we present a method that combines the power of high-content single cell measurements with the efficiency of high-throughput techniques. A calibration on the basis of identical cell populations measured by both approaches connects the two techniques. We develop a mathematical model to relate quantities exclusively observable by high-content single cell techniques to those measurable with high-content as well as high-throughput methods. The latter are defined as free variables, while the variables measurable with only one technique are described in dependence of those. It is the combination of data calibration and model into a single method that makes it possible to determine quantities only accessible by single cell assays but using high-throughput techniques. As an example, we apply our approach to the nucleocytoplasmic transport of STAT5B in eukaryotic cells.

**Conclusions:**

The presented procedure can be generally applied to systems that allow for dividing observables into sets of free quantities, which are easily measurable, and variables dependent on those. Hence, it extends the information content of high-throughput methods by incorporating data from high-content measurements.

## Background

In systems biology, a wide range of experimental data is used for mathematical modeling. Qualitative data mostly serves as a basis for determining network structures, whereas dynamic pathway modeling relies on high-quality quantitative data. In general, experimental data describing biological systems can be divided into three groups. Firstly, data generated from large cell populations yields an average information of the whole population behavior. However, cell population assays such as biochemical measurements or microarray studies can be misleading as large cell-to-cell variations are often observed, even in seemingly uniform populations. This stochasticity can be caused by asynchronous cell cycles, differences in cell sizes and varying protein states or expression levels [1-3]. Secondly, single cell data with high-content information from a limited number of cells result in a stochastic distribution of measured quantities. Many single cell approaches are based on microscopy, but other technologies are under development to investigate for example gene expression or proteins in single cells [4-6]. The third group covers a small range of experimental techniques that generate single cell data from large cell populations in a high-throughput format. Most common among those is flow cytometry, which however is limited to measurements from cells in suspension. Moreover, in contrast to microscopy, standard flow cytometry can only detect average whole cell fluorescence intensities lacking spatially resolved information. Currently, high-throughput imaging techniques as well as imaging flow cytometers digitally imaging cells directly in flow are being developed, with the goal to gather high-content information from a large number of single cells [7,8]. This will increase the number of parameters that can be determined in parallel by high-throughput and high-content techniques.

For modeling purposes it is essential to link data from different types of experiments in order to include as many details of the system as possible in the modeling process and to avoid non-identifiabilities during the parameter estimation. However, some of the components can only be measured by time consuming high-content techniques. For models describing entire cell populations, high-content data for large cell numbers is necessary but often impossible to provide. In contrast, high-throughput techniques can generate these large data sets, despite a lack in detailed single cell information.

A signaling pathway that has been extensively investigated by dynamic pathway modeling is the JAK-STAT pathway [9]. Upon binding of an extracellular ligand to the respective receptor latent signal transducers and activators of transcription (STATs) are activated by Janus kinases (JAK) leading to rapid nucleocytoplasmic cycling of STATs. In addition, constitutive nucleocytoplasmic cycling of unphosphorylated STAT has been shown for several STAT proteins by biochemical and microscopic experiments [10-15]. It has been proposed that import of STAT is enhanced upon activation [16], while export of activated STAT is slowed down either through retention in the nucleus by DNA binding [17] or possibly a different export mechanism [15]. Previously, rapid nucleocytoplasmic cycling of activated STAT5 has been identified as the step most sensitive to perturbation within the core module of the JAK2/STAT5 pathway by mathematical modeling based on biochemical data [18], but import and export rates could not be measured experimentally. These transport steps are crucial as important decisions regulating cell fate are made by the nuclear reactions of STATs.

A method to determine the rates for nuclear import and export of STAT5 is fluorescence recovery after photobleaching (FRAP). FRAP is a single cell fluorescence microscopy method routinely used to measure the kinetics of transport processes between cell compartments as well as diffusion and dynamic binding reactions [19,20]. One prerequisite for a quantitative FRAP experiment is that the investigated system is in a steady state on the time scale of the experiment otherwise a mathematical description of the data is difficult to obtain. The JAK-STAT system is only in a steady state in unstimulated cells, ligand stimulation induces phosphorylation of STATs and thereby perturbs the steady state. Therefore, we focused on the nuclear import and export rates of unphosphorylated STAT5 with the goal to generate rates for the steady state in unstimulated cells that can be set to a fixed value in a larger pathway model. Biochemical data describing the phosphorylation dynamics of the pathway components after stimulation in combination with mathematical modeling can then serve to indirectly determine nuclear import and export rates for phosphorylated STAT5.

Here, we present a model for extracting the import and export rates from FRAP experiments of STAT5B-GFP in the steady state of unstimulated NIH3T3-EpoR cells. Furthermore, the dependence of these rates on STAT5B-GFP concentration and cell size is shown. To be able to combine this information with biochemical data from cell populations expressing STAT5B-GFP, cell size distribution and STAT5B-GFP concentration distribution within the respective cell population are additionally measured by flow cytometry. Cell size as well as STAT5B-GFP concentration are estimated directly from flow cytometry data after calibration of these data to microscopy data.

The calibration procedure can be generally applied to link data from powerful high-content techniques and fast, efficient high-throughput methods. In combination with the mathematical model, it provides a novel rationale to determine formerly inaccessible information for large cell populations by less time-consuming high-throughput measurements.

## Results and Discussion

### Data calibration links high-content with high-throughput data

To formulate a general calibration procedure for combining high-content and high-throughput data we use a method based on *least squares regression of the quantile-quantile plot *(QQ-plot) for corresponding population measurements. Let

(1) $$\text{High-Content}:Y=mX_{C}+d$$

(2) $$\text{High-Throughput}:Y=m^{\prime }X_{T}+d^{\prime }$$

where *Y *refers to the quantity of interest, e.g. protein concentration, cell volume, total amount of protein, *X*_*C *_and *X*_*T *_are observables for the high content or high-throughput technique that are both linearly connected to *Y *via slopes *m*, *m' *and intercepts *d, d'*. In a more general formalism, a measurement technique is a strictly monotonic function *F *with *Y = F*(*X*), i.e. *F *uniquely relates an observable to a quantity of interest. Practically, the scale of *X *is chosen in such a way that *F *is linear. The strict monotony of *F *requires *m*, *m' *to be non-zero. Eqs. (1) and (2) show that for every value of *Y*

(3) $$mX_{C}(Y)+d=m^{\prime }X_{T}(Y)+d^{\prime }$$

(4) $$\Leftrightarrow X_{T}(Y)=\frac{m}{m^{\prime }}X_{C}(Y)+\frac{d-d^{\prime }}{m^{\prime }},$$

i.e. *X*_*T *_(*Y *) depends linearly on the high-content quantity *X*_*C*_(*Y *). The slope $\frac{m}{m^{\prime }}$ and intercept $\frac{d-d^{\prime }}{m^{\prime }}$ of eq. (4) need to be determined in order to translate *X*_*C *_into *X*_*T *_and vice versa. For this purpose, the distribution quantiles of *X*_*C *_and *X*_*T *_are used.

Assuming that *N*_*C *_and *N*_*T *_>*N*_*C *_measurements have been performed for the high-content and high-throughput techniques, respectively, the ordered set of measurements {*X*_*C,i*_}_*i *= 1_,...,*N*_*C *_is an estimate of the *N*_*C *_equally spaced quantiles ${\left\{{\tilde{X}}_{C,i}\right\}}_{i=1,...,N_{C}}$ of the theoretical distribution of *X*_*C*_. In the same way, the sample quantiles ${\left\{{\tilde{X}}_{T,i}\right\}}_{i=1,...,N_{C}}$of {*X*_*T,i*_}_*i *= 1_,...,*N*_*T *_estimate the *N*_*C *_theoretical quantiles of *X*_*T*_. According to eq. (4) the distributions of *X*_*T *_and *X*_*C *_belong to the same location-scale family. Consequently, the QQ-plot of *X*_*T *_versus *X*_*C *_is supposed to follow a straight line with intercept $\frac{d-d^{\prime }}{m^{\prime }}$ and slope $\frac{m}{m^{\prime }}$. A least squares fit of the QQ-plot gives asymptotically unbiased estimates of slope and intercept for a large class of theoretical distributions. The convergence of the sample quantiles to the theoretical quantiles as well as the convergence of the least squares estimator is well known and is carried out rigorously in [21].

An implementation of this calibration procedure is provided by the R script [Additional file 1] in the supplement. A sample configuration is given by [Additional file 2].

**Figure 1:**
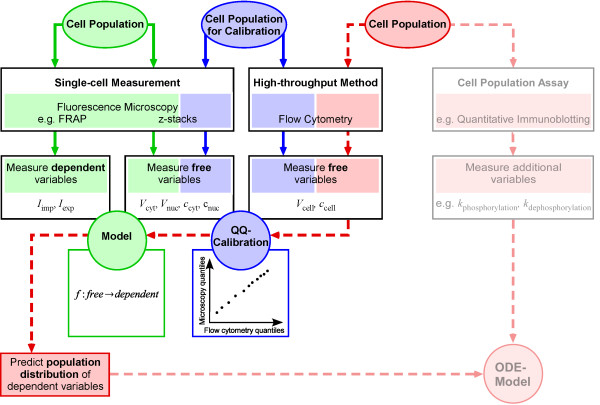
**Workflow for calibrating and linking data from different experiments**. The calibration and model are the crucial steps of the procedure described here. Data calibration by a quantile-quantile (QQ) plot allows to translate data from one technique to the units of another measurement. Here, it is necessary to determine quantities with a known relationship from the identical cell population by both methods (blue). Furthermore, a model has to be developed to describe the dependent variables only measurable by one technique in terms of the free variables assessable by both techniques (green). Then, additional cell populations can be measured by the high-throughput technique only and the information content of the data can be increased via the calibration and the model (dashed arrows, red) to be finally combined with cell population data for mathematical modeling by an ordinary differential equation (ODE) model (transparent red). General terms are shown in bold letters, the specific case of the example system is given below. Steps shown in transparent colors are not subject of this study.

The calibration is then included in the overall procedure linking high-throughput with high-content data (fig. 1). The essential steps are the calibration and the mathematical model depicted in the center of the workflow diagram. For calibration, the identical cell population is measured by a single-cell technique as well as a high-throughput method to determine a subset of matching quantities, defined as the free variables. At the current state of technology only flow cytometry is widely available and fulfills the requirement of generating high-throughput data at the single-cell level. This restricts the free variables that can be determined experimentally to cell volume (*V*_*cell*_) and concentration of a fluorescently labeled marker (*C*_*cell*_). A high-content technique that can be combined with flow cytometry and that can also assess cell volume and fluorescence intensity is microscopy. As new high-throughput techniques advance other parameters can be considered as free variables. The data for the free variables are then compared and the resulting calibration creates the possibility to switch between the different measurement units. Next, the quantities of interest, i.e. any high-content information determined by for example microscopy that is dependent on cell size or concentration of the fluorescently labeled marker or both, need to be expressed in dependence of the free variables. A valid model and parameter estimation connecting dependent and free variables of the single cell measurement has to be identified. The high-throughput measurements can then be translated via calibration into the ambit of single-cell measurements and via the fixed parameter model into cell population quantities. The method can be applied to combine experimental data generated by different experimental techniques if the free variables can be measured by all of the techniques used for data generation.

### Nucleocytoplasmic cycling of STAT5B is modeled as saturatable pump

As an example, the analysis of nuclear import and export of the transcription factor STAT5B was chosen. Nucleocytoplasmic cycling is only measurable by single cell microscopy, namely FRAP, whereas other crucial features such as the dynamic changes of the phosphorylation state of the proteins are accessed by biochemical measurements from cell populations. In eukaryotic cells, the nucleus is separated from the cytoplasm by the nuclear envelope. Molecules can only migrate between those two compartments through nuclear pores forming small holes in the membrane. Small molecules (<20-40 kDa) can diffuse freely through nuclear pores whereas larger molecules require active transport aided by soluble transport proteins that interact with the cargo molecule as well as the nuclear pore. Active nuclear import and export are regulated by different mechanisms. In order to be imported into the nucleus, proteins usually carry a nuclear localization signal (NLS) to which importins can bind and enable nuclear translocation. Similarly, a nuclear export signal (NES) within the cargo protein structure is recognized by an exportin. For most proteins of the STAT family, the respective importins have been identified (reviewed in [22]). In the case of STAT5B however, so far no importins could be identified that directly interact with the transcription factor [23]. Instead, import of STAT5B has been suggested to require additional factors acting as chaperones between the importins and STAT5B [24]. Active nuclear export of STATs is generally mediated by the exportin CRM1.

Here, a simple model for the active transport of STAT5B through the nuclear pore was used. A single nuclear pore and the respective import and export factors necessary to transport a single protein of interest were modeled as a pump [25,26] making the following assumptions: For small concentrations, the amount of protein transported through the pores is proportional to the concentration. For large concentrations the transport current, i.e. the number of molecules per time, saturates. For a large set of nuclear pores for which the capacity of every pore may vary a *Michaelis-Menten *curve is a reasonable way to model the overall current.

(5) $$I(c):=\dot{N}=\frac{\beta c}{\gamma +c}.$$

For large concentrations (*c *≫ *γ*) *I *saturates with saturation value *β*. For small concentrations (*c *≪ *γ*) *I *depends linearly on *c *with slope $\frac{\beta }{\gamma }$.

Equation (5) is the resulting current for all pores of a cell. The constants *β *and *γ *may still vary within a population, i.e. from cell to cell. In a next step the saturation value *β *= _*k*_*K *as well as the slope $\frac{\beta }{\gamma }=\kappa^{\prime }K$ are assumed to depend linearly on a quantity *K *which is the product of the abundance of transport factors and the number of nuclear pores. This is appropriate for two reasons:

First, if the system is in saturation and the number of nuclear pores is doubled, then the system has twice the capacity to transport STAT5B and the current will be doubled. The same holds for the transport factors. Hence, the saturation value *β *is proportional to the product of transport factor abundance and nuclear pore number. Second, if the STAT5B concentration is low and consequently the transport rate is independent of the concentration then doubling the number of pores or the number of transport factors will lead to a doubling of the transport rate. Hence, the transport rate, i.e. the slope of the current $\frac{\beta }{\gamma }$, is proportional to the product as well. Plugging in *β *= *κK *in $\frac{\beta }{\gamma }=\kappa^{\prime }K$ reveals that $\gamma =\frac{\kappa }{\kappa^{\prime }}$ is independent of *K *and eq. (5) reads

(6) $$I_{K}(c)=\frac{\kappa Kc}{\frac{\kappa }{\kappa^{\prime }}+c}.$$

Consequently, given an arbitrary cell from the population and knowing about *K*, the current *I*_*K *_is a much better estimator for the transport current than the mere population average. The question arising from this is if and how *K *is accessible. Three cases seem plausible:

1. *K *is dominated by the number of nuclear pores which have a similar density throughout the cell population. Hence, *K *depends linearly on the **nuclear surface area ***A*_*nuc*_.

2. *K *is dominated by the number of cytoplasmic transport factors with the same concentration in all cells which is proportional to the **cytoplasmic volume ***V*_*cyt*_.

3. *K *is dominated by the number of nuclear transport factors with the same concentration in all cells which is proportional to the **nuclear volume ***V*_*nuc*_.

The different hypotheses represent different models, model 1 is without any normalization. Models 2 - 4 are defined by the respective normalized currents:

(7) $$j_{A_{nuc}}=\frac{I(c)}{A_{nuc}},\;j_{V_{nuc}}=\frac{I(c)}{V_{nuc}}\;\text{and}\;j_{V_{cyt}}=\frac{I(c)}{V_{cyt}}.$$

Reformulating the problem as

(8) $$j_{l}(c)=\frac{\beta_{l}c}{\gamma_{l}+c}\equiv \alpha_{l}(c)\;\cdot \;c,\;l=A_{nuc},\;V_{nuc},\;V_{cyt}$$

demonstrates the concentration dependency of the normalized transport currents under the assumption that the parameters *β*_*l *_and *γ*_*l *_are constant throughout the population. This assumption is necessary for a valid formula describing the import and export currents within a population. The second formulation with *α*_*l *_(c) follows the idea of a linearly increasing current for small concentrations and will also be used.

### Import and export current distribution for STAT5B

#### Import and export currents depend on STAT5B concentration and cell size

To determine the import and export rates *α*_*imp *_and *α*_*exp *_39 FRAP data sets generated from cells expressing varying concentrations of STAT5B-GFP were fitted with eq. (23) described in the Methods section. Variable protein levels were achieved by a tightly regulatable expression system that we developed based on a Tet-inducible promoter.

**Figure 2:**
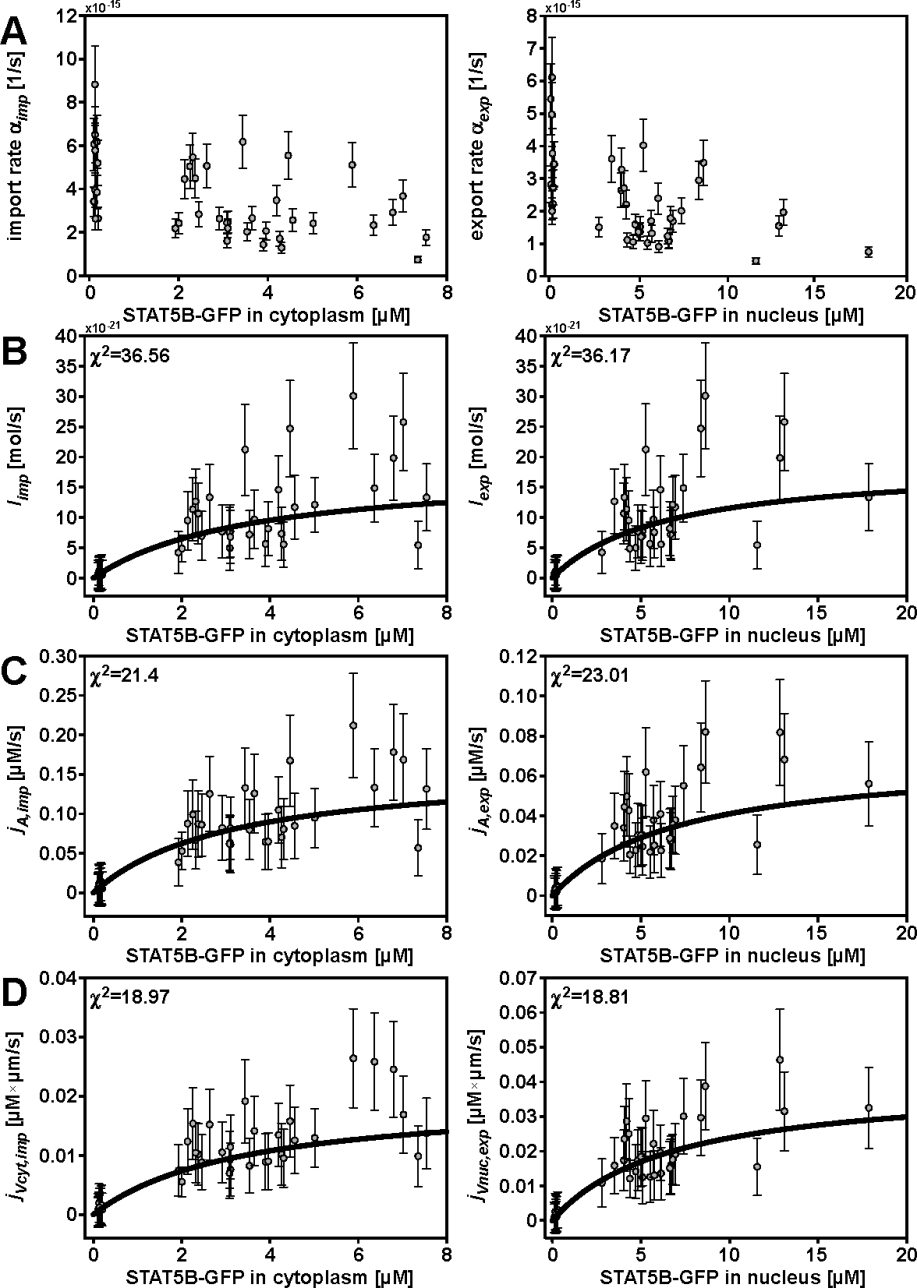
**Rates and currents of STAT5B nucleocytoplasmic cycling**. (A) Summary of all rates *α*_*imp *_and *α*_*exp*_. The rates directly correspond to the exponential of the fits to the FRAP data. Gaussian error propagation leads to the error bars for *α*_*imp *_and *α*_*exp*_. The relative uncertainty of the fitted parameter *a*_1 _is negligible compared to the relative uncertainty of the denominator. Relative errors of concentrations and volumes can be estimated to be around 10% and lead to the rate uncertainties. In addition, for the currents (panels (B)-(D)) a small constant error has been added to every point to avoid overvaluing small currents. (B) Michaelis-Menten fit for import (left) and export currents (right) not normalized, (C) normalized to nucleus surface area and (D) normalized to the respective originating compartment volume. χ^2 ^values of the fit are indicated in the plot.

The cell-to-cell variability of *α*_*imp *_and *α*_*exp *_exceeded the confidence intervals of the rate values by far (fig. 2A). This supported the assumption of a confounding variable *K*. The three hypotheses described above were tested for cell-to-cell variability of the transport rates. The comparison of different normalizations was based on the normalized currents *j*_*l *_(*c*) given by equation (8). For every normalization the Michaelis-Menten curve was determined from a least squares fit, i.e. the different normalizations were ranked by decreasing χ^2 ^values. The results for no normalization, normalization by the nuclear surface area and normalization by the originating compartment volumes are shown in fig. 2B-D, the estimated parameters are shown in tab. 1.

**Table 1:** Estimated Parameters

	*β *[mol/s]	*γ *[*μ*M]
*I*_*imp*_	18.04 ± 6.55	3.56 ± 2.63

*I*_*exp*_	19.01 ± 7.06	6.49 ± 4.71

	*β*_*A *_[mM/s]	*γ *[*μ*M]

*J*_*imp,A*_	16.13 ± 3.88	3.12 ± 1.63

*J*_*exp,A*_	74.72 ± 22.21	7.07 ± 3.96

	*β*_*V *_[mM *μ*/s]	*γ *[*μ*M]

$J_{imp,V_{nuc}}$	86.37 ± 18.82	2.98 ± 1.44

$J_{exp,V_{nuc}}$	40.13 ± 10.54	6.81 ± 3.42

$J_{imp,V_{cyt}}$	22.14 ± 5.30	3.43 ± 1.71

$J_{exp,V_{cyt}}$	8.28 ± 2.37	5.31 ± 3.25

Estimated parameters *β*, *β*_*A*_, *β*_*V *_and *γ *from the χ^2 ^fit. The uncertainties correspond to a 1σ confidence level.

**Figure 3:**
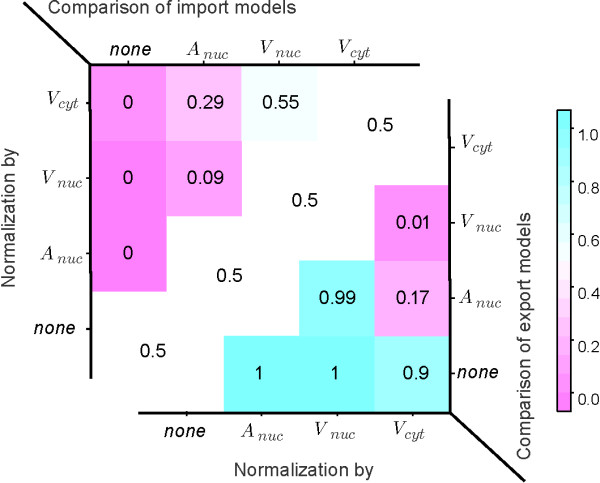
**Pairwise import/export model comparison**. Plot of the estimated significance of model difference for pairwise compared import models (upper left triangle) and export models (lower right triangle). Values *q *< 0.5 indicate superiority of the model on the vertical axis compared to the model on the horizontal axis at a confidence level of 1 - *q*. Accordingly for values *q *> 0.5.

The significance of the χ^2 ^reduction has been tested with a bootstrap method: from the 39 data points 39 points have been drawn randomly with replacement. Then for all models, i.e. without normalization, *K *∝ *A*,*K *∝ *V*_*nuc *_and *K *∝ *V*_*cyt *_pairwise differences $\delta_{ij}=\chi_{i}^{2}-\chi_{j}^{2}$of the χ^2 ^values have been computed leading to 6 difference values for the import and 6 difference values for the export models. This procedure has been repeated 10^4 ^times resulting in 2 × 6 distributions $\delta_{ij}^{\left(exp\right)}$ and $\delta_{ij}^{\left(imp\right)}$of χ^2 ^difference values. The position of zero with respect to such a distribution decides whether one of the compared models is superior to the other. More precisely:

Let $q_{ij}^{\left(exp\right)}=P_{\delta_{ij}^{\left(exp\right)}}(\delta <0)$ be the probability that a value *δ *drawn from the distribution $\delta_{ij}^{\left(exp\right)}$ is lower than zero. Then $q_{ij}^{\left(exp\right)}<p$ means that export model *j *is superior to export model *i *at a confidence level of 1 - *p*. The other way round, $q_{ij}^{\left(exp\right)}>(1-p)$ means that model *i *is superior to model *j *at 1 - *p *confidence level. Analogously for *imp*. The computed values $q_{ij}^{\left(imp\right)}$ and $q_{ij}^{\left(exp\right)}$ can be found in fig. 3, import values in the upper left triangle, export values in the lower right triangle.

For the export distributions model 3 - normalization by nucleus volume - is superior to all other models at a 99% confidence level (3σ). For the import data the situation is not so clear. Models 3 and 4 cannot be discriminated and seem to describe the data equally well. Both models are clearly superior to model 1 and exceed model 2 at a 1σ level.

We decided to follow the hypothesis of normalization by the volumes of the originating compartments, i.e. export model 3 and import model 4. From a biological point of view this seems to be the most reasonable hypothesis. From a practical point of view, models 3 and 4 describe the import equally well and cannot be distinguished given the data at hand.

#### Calibration of flow cytometry data to microscopy data yields comparable quantities

**Figure 4:**
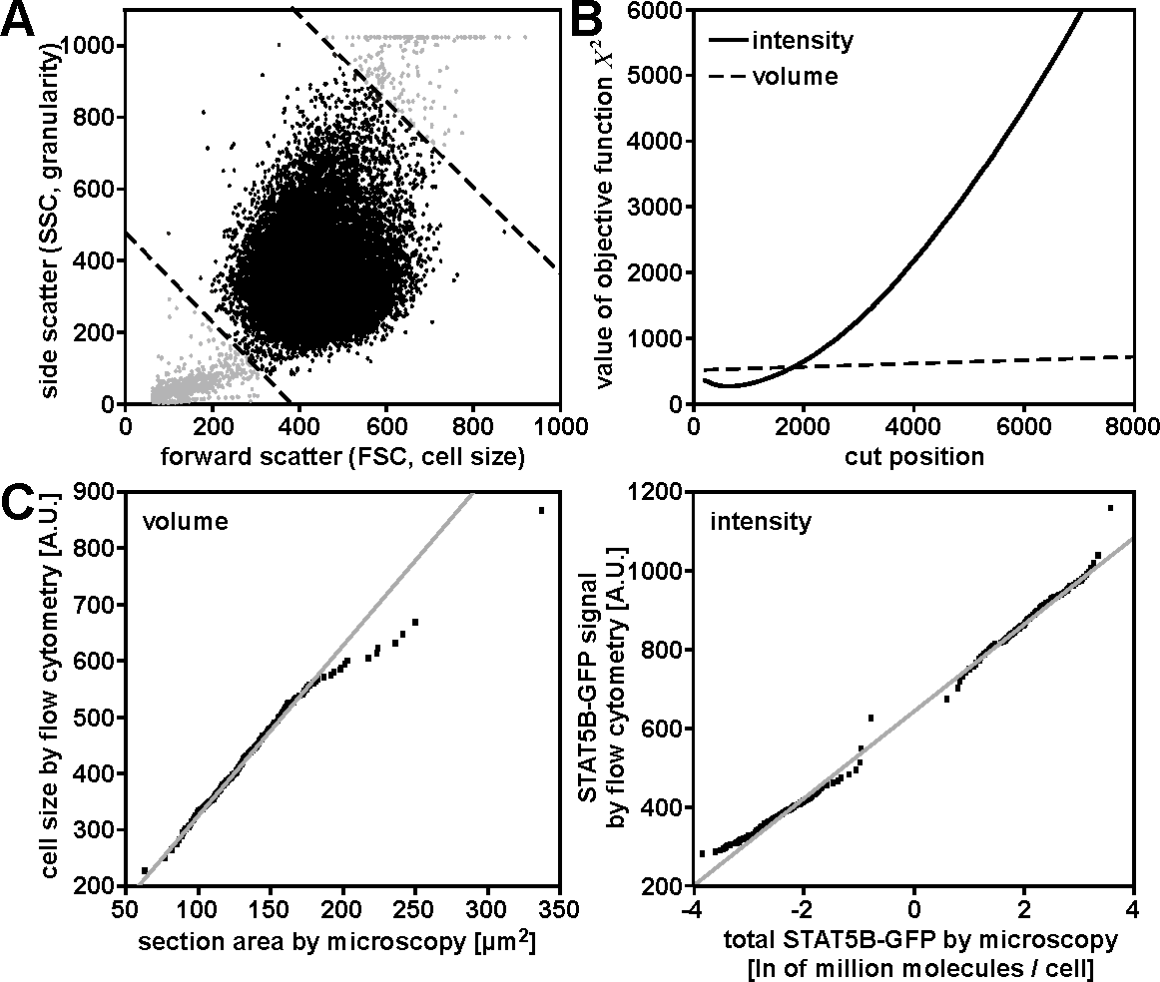
**Data calibration**. (A) Scatter plot of flow cytometry forward scatter versus side scatter. Excluded data points are shown in light grey. Dashed lines indicate chosen cut for data exclusion. (B) *X*^2^curves for the quantile-quantile plot versus the cut position for the flow cytometry data for cell volume (dashed line) and fluorescence intensity (solid line). (C) Quantile-quantile plots for cell volumes (left) and fluorescence intensities (right) used for calibration. Cell populations treated with 10 ng/ml and 250 ng/ml doxycycline are both included. Flow cytometry data are raw data, microscopy data have been transformed to represent the same parameters as flow cytometry data. The number of quantiles corresponds to the number of microscopy data points.

Data calibration requires the measurement of the identical cell population by both techniques. In con-focal microscopy only intact, living cells attached to a surface can be observed. However, for flow cytometry cells are detached from their growth surfaces, generating a cell suspension of intact, living cells mixed with dead cells and cell fragments. Therefore, the flow cytometry data have to be preprocessed, so that it only includes living cells and is comparable to the microscopy data. To achieve this, the scatter (*F*_0 _∝ cell cross-section area) was plotted against the side scatter (*F*_1 _∝ granularity) (fig. 4A). To exclude dead cells and cell fragments, linear cuts were sufficient: a line through the point cloud was defined by linear regression without intercept for the scatter plot. Based on this line two perpendicular lines - the cuts - were introduced separating vital cells in the inner region from undesired cells in the outer region. The resulting subset of cells had the same volume and STAT5B-GFP distribution as the microscopy cells.

To yield comparable distributions, it has to be considered that different quantities are measured by the two techniques. Microscopy data directly result in absolute numbers for compartment volumes and protein concentrations, whereas flow cytometry data from the fluorescence intensity channel *F*_2 _are logarithmic due to the amplification of the signal by the instrument and the forward scatter of a flow cytometer using light scattering is an approximate measure of the cell cross-section area (see [27]). Therefore, values from either flow cytometry or microscopy measurements had to be transformed to yield comparable quantities. For practical reasons, the microscopy fluorescence intensities *I*_*micro *_were logarithmized yielding *X*_*M *_= log *I*_*micro*_. Similarly, cell volumes *V*_*micro *_determined by microscopy were converted to cross-section areas assuming a spherical shape of the cell as is the case for flow cytometry samples. This yields $X_{M}=\sqrt[{3}]{36\pi }\;\cdot \;V_{micro}^{\frac{2}{3}}$. For flow cytometry, *X*_*F *_= *F*_2 _and *X*_*F *_= *F*_0 _for fluorescence intensity and cross section area respectively. *X*_*M *_and *X*_*F *_defined like this build the basis for the calibration method described above.

To take the different sensitivities of the two experimental techniques for fluorescence detection into account, the flow cytometry data was corrected for cells that are too dim to be detected by microscopy. Then, the quantiles of *X*_*M *_were plotted versus the quantiles of *X*_*F. *_The best accordance in the QQ-plot is expected if an additional cut-off is introduced to the flow cytometry data: only *X*_*F *_>*Z *for a cut-off parameter *Z *is taken into account. The accordance is measured by summing up al l squares of the residuals for the QQ-plot (absolute χ^2^) and *Z *is chosen optimally if χ^2 ^reaches a local minimum. In order to avoid that *Z *is chosen too large and large parts of the flow cytometry distributions are dropped, large *Z *values were penalized quadratically. The objective function is

(9) $$X^{2}=\frac{1}{2}\chi^{2}+\frac{1}{2}\chi^{2}(n+1)p^{n}$$

where *p *∈ 0[1] is the fraction of the population that is dropped and *n *= 2 for quadratic penalization. The penalization is chosen on purpose to fulfill ⟨(*n *+ 1)*p*^*n*^)⟩ = 1 for uniformly distributed *p*. This guarantees that the penalization is of the same magnitude as χ^2^. The resulting *X*^2 ^curve for the size distribution indicated that only the complete flow cytometry data set lead to the best accordance, while a local minimum existed for the fluorescence intensity distribution (fig. 4B).

After choosing the optimal cut-off, a least squares regression was applied to the QQ-plot. The linearity of the data points confirmed that the shapes of the two distributions are the same. However, even after two cuts there were deviations for the border points that result from a small population of cells which is detected differently by flow cytometry and by microscopy. To exclude biased fit parameters the least squares regression was restricted to the inner 66% region of points (fig. 4C).

Thus, data preprocessing and subsequent least squares regression of the QQ-plot lead to comparable quantities obtained by different experimental techniques. All functions for preprocessing the flow cytometry data and for calibration of flow cytometry to microscopy data are included in the R script 3.1 provided in the supplement.

#### Distributions of transport currents for an exemplary cell population are calculated

In order to compute the distribution of currents for a sample flow cytometry measurement, the calibration was combined with the formula describing the currents (eq. (8)). As has been shown above (fig. 2D), the rates *α*_*in *_and *α*_*out *_depend on the STAT5B concentrations *C*_*cyt *_in the cytoplasm and *C*_*nuc *_in the nucleus as well as the compartment volumes *V*_*nuc *_and *V*_*cyt *_for nuclear export and import, respectively.

Since the individual cell compartments cannot be distinguished by flow cytometry, an average ratio of the cytoplasmic to nuclear quantities had to be estimated from microscopy data. For every FRAP data set, the fractions $f_{V}=\frac{V_{cyt}}{V_{nuc}}$ and $f_{c}=\frac{c_{cyt}}{c_{nuc}}$ were determined and averaged. In addition, we tested if the fraction *f*_*V *_and the cell volume *V *or *f*_*C *_and the total concentration *c *of STAT5B-GFP are correlated. A large correlation value would indicate that an additional model for describing the dependency of the compartment quantities on the overall quantities would be necessary. The data lead to *f*_*V *_= 4.27 ± 0.11 and *f*_*c *_= 0.645 ± 0.015 and the correlation test revealed cor(*f*_*V*_, *V *) = 0.15 ± 0.28 and cor(*f*_*c*_, *c*) = -0.37 ± 0.26. Thus, the assumption of a correlation for the cell volume would not lead to a better estimate of *f*_*V *_Even for *f*_*c *_considering the correlation would have a minor effect.

**Figure 5:**
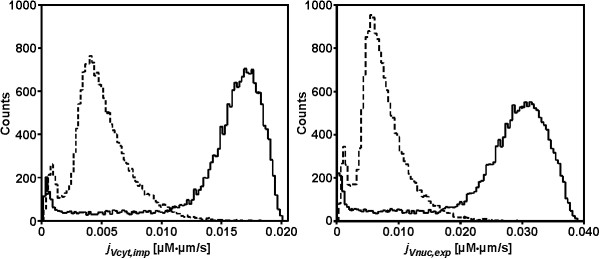
**Current distribution**. Distribution of import (left) and export (right) currents for exemplary cell populations treated with 5 ng/ml doxycycline (dashed lines) or 50 ng/ml doxycycline (solid lines). Transport currents are normalized to the size of the respective originating compartment.

For the population current calculation, only the mean values of *f*_*V *_and *f*_*c *_were used. The resulting distributions of import and export currents are shown in fig. 5. The transport currents were determined for two cell populations expressing either very low or very high levels of STAT5B-GFP. For import as well as export currents the distributions show different average values but similar variance. This current distribution can be directly combined with other population data generated from the same cell population, such as biochemical time course data describing the phosphorylation dynamics of the proteins involved. Only by using both types of data for mathematical modeling it is possible to combine a detailed experimental investigation of nuclear import and export with signal transduction mediated by phosphorylation of signaling proteins.

## Conclusion

In this study, transport rates for unphosphorylated STAT5B were determined in single cells by FRAP and found to follow saturation kinetics dependent on both STAT5B-GFP expression level as well as size of the originating compartment. This reflects a saturation of cofactors necessary for active transport of STAT5B through the nuclear pore complex. The parameters for concentration and volume dependency of the cycling currents were estimated. To predict transport currents with the saturation model for large cell populations, STAT5B-GFP concentration and cell size distribution were measured by flow cytometry. As flow cytometry only yields relative values for cell size and total cell fluorescence, a calibration to absolute numbers generated by single cell microscopy is required. For calibration, the concentration of the transcription factor STAT5B as well as cell size were determined by confocal microscopy and flow cytometry from the identical cell population, resulting in a linear calibration curve. Subsequently, absolute cell size and STAT5B-GFP concentration distributions were computed from flow cytometry data using the calibration curve. Finally, transport current distributions and thereby cell-to-cell variation were predicted using the saturation model.

In recent years, other members of the STAT protein family have been studied by FRAP to investigate nucleocytoplasmic cycling [12,15,28], but the data have so far only been interpreted qualitatively. Our results provide a procedure to link directly measured import and export rates of unphosphorylated STAT5B with data indirectly describing the nucleocytoplasmic cycling of activated STAT5B generated by biochemical experiments. Furthermore, by using an inducible expression system for STAT5B-GFP, we identify a saturation-like behavior of STAT5B nuclear import and export, indicating a limitation in transport factors. The nature of these factors remains to be identified.

The proposed method is generic and is applicable as long as two conditions are fulfilled. First, the quantities that are measured by a certain high-content method have to be functionally related. This allows for expressing a subset of the quantities, defined as the dependent variables, as a function of the remaining, free variables. Second, the free variables have to be part of the quantities that can be measured by a given high-throughput method. If these conditions hold, it is possible to determine the function connecting free and dependent variables by setting up a mathematical model and estimating its parameters. Furthermore, it is possible to calibrate the two measurement techniques against each other as the high-throughput quantities are in particular part of the high-content quantities. This means that a high-throughput measurement can be translated into the ambit of a high-content measurement. Applying the fixed parameter model then leads to a prediction of the dependent variables' distributions representing an indirect determination of these variables for every cell of the population. The method is especially useful if there is a great discrepancy in accessibility between free and dependent variables. This combination of two experimental approaches results in a higher degree of measured variables suitable for mathematical modeling and a reduction of non-identifiabilities in the parameter estimation.

## Methods

### Experimental Procedures

The retroviral expression vector pMOWS containing the cDNA for murine HA-EpoR was introduced into NIH3T3 cells (ATCC) and a single cell clone stably expressing HA-EpoR was obtained by selection with G418. pMOWSIN-TREt-STAT5B-GFP was cotransduced into NIH3T3-EpoR cells together with the cDNA for the transactivator protein contained in pMOWS-rtTAM2. A single cell clone stably expressing murine STAT5B-GFP was obtained by selection with puromycin. Expression of STAT5BGFP was regulated by a Tet-inducible promoter included in pMOWSIN-TREt. pMOWSIN-TREt was generated by digesting pTRE-tight (Clontech) and inserting TREt into the self-inactivating (SIN) retroviral vector pMOWSIN. pMOWS-rtTAM2 was generated by introducing cDNA of rtTAM2 from pUHrT-62-1 (H. Bujard, Heidelberg, Germany) into pMOWS using BamHI/EcoRI restriction sites [29]. To simplify identification of the nuclei cells used for FRAP experiments also were transduced with pMOWS-H2B-mCherry. All cells were maintained in DMEM supplemented with 10% calf serum and 1% PenStrep.

For FRAP experiments cells were grown to 60-80% confluency in Labtek chambered coverglasses over night. Doxycycline was added at a concentration of 10-250 ng/ml approximately 16 hours before serum-starvation. Cells were serum-starved in DMEM supplemented with 25 mM HEPES pH 7.4 and doxycycline for at least 5 hours. Confocal microscopy was performed on a Leica SP5 with a 63×/1.4 NA oil immersion objective and the pinhole set to 1 Airy unit. All live cell imaging was performed at 37°C. For cell volume and STAT5B-GFP concentration estimation a *z*-stack of the entire cell was acquired prior to each FRAP experiment. Cytoplasmic and nuclear volume was estimated from *z*-stack data by measuring the whole cell area or nuclear area of each slice in ImageJ (see [30]) and calculating the respective volume by summing up the voxels per slice. Cytoplasmic volume *V*_*cyt *_was calculated as the difference of the nuclear volume *V*_*nuc *_subtracted from the whole cell volume *V*_*cell*_. To avoid overestimation of bright cells due to scattered light, the number of slices considered was determined by measuring the maximum average intensity in a small region of the nucleus over the whole stack. Only those slices with at least half the maximum mean intensity were included in the analysis. Mean intensities in the nucleus and the whole cell were converted to GFP concentrations by using a dilution series of recombinant SBP-GFP in PBS and embedded in 15% polyacrylamide gel as reference. The cytoplasmic concentration of STAT5B-GFP *c*_*cyt *_was calculated as

(10) $$c_{cyt}=\frac{V_{cell}c_{cell}-V_{nuc^{c}nuc}}{V_{cyt}}$$

To determine import and export currents, STAT5B-GFP was photobleached in the entire nuclear region with 100% laser power (488 nm). For analysis of the transport dynamics 10 prebleach and approximately 240 postbleach images of the whole cell were acquired for 30-40 min after bleaching.

Flow cytometry analysis of STAT5B-GFP expression level and measurement of the approximate cell size were performed on a BD FACSCalibur system with the software package CellQuest. Cells were grown in 60 mm cell culture dishes and were treated as for microscopy. Cells were detached from the dishes by 0.05% trypsin/EDTA and washed once in PBS/0.3% BSA. For each cell population 20 000 cells were measured. Forward and side scatter were detected linearly, for fluorescence intensity detection the signal was logarithmically amplified. NIH3T3-EpoR cells were used as control for cellular autofluorescence and cell size. Raw data was extracted from CellQuest files with FCSExtract [31]. Fluorescence intensity values were directly used for analysis. Values for the forward scatter were assumed to be approximately proportional to the cross-section area of the cell [27]. Cell shape was assumed to be roughly spherical for detached cells and therefore the relation between cross-section area and volume is known.

For the calibration measurement, cells from one cell population were seeded in 60 mm dishes as well as Labtek chambers 20 hours before the experiment. STAT5B-GFP expression was induced with either 10 or 250 ng/ml doxycycline 16 hours prior to serum-starvation. Flow cytometry analysis was performed as described above. *z*-stacks of 100 tiled frames were acquired by confocal microscopy. For each doxycyline treatment the cell volume and the total amount of STAT5B-GFP per cell were determined for 200 cells as described for FRAP experiments above.

### Mathematical Model

#### Import and export currents from FRAP data

In the biological system employed here, fluorescently labeled STAT5B (STAT5B-GFP) is introduced into the cells in addition to endogenous STAT5B so that

(11) $$c_{cyt}=c_{cyt,L}+c_{cyt,E}$$

(12) $$c_{nuc}=c_{nuc,L}+c_{nuc,E}$$

consist always of the sum of labeled (*L*) and endogenous (*E*) molecule concentrations. With the concentrations normalized currents are associated:

(13) $$j_{imp,L}=\frac{c_{cyt,L}}{c_{cyt}}j_{imp}$$

(14) $$j_{exp,L}=\frac{c_{nuc,L}}{c_{nuc}}j_{exp}.$$

Here *imp *indicates transport from cytoplasm to nucleus and accordingly *exp *transport from nucleus to cytoplasm. From the definition of *j*_*imp/exp *_arises a system of coupled differential equations for the labeled molecule concentrations:

(15) $${\dot{c}}_{cyt,L}=-\frac{Kj_{imp}}{V_{cyt^{c}cyt}}c_{cyt,L}+\frac{Kj_{exp}}{V_{cyt^{c}nuc}}c_{nuc,L}-\epsilon c_{cyt,L}$$

(16) $${\dot{c}}_{nuc,L}=\frac{Kj_{imp}}{V_{nuc^{c}cyt}}c_{cyt,L}-\frac{Kj_{exp}}{V_{nuc^{c}nuc}}c_{nuc,L}-\epsilon c_{nuc,L}.$$

As in the previous section *K *= *A*, *V*_*cyt*_, *V*_*nuc *_accounts for the normalization. The associated index *l *is omitted as an index of *j*. The ε-terms describe the continuous bleaching due to constant laser exposition during postbleach image acquisition.

The two-compartment system is in equilibrium, i.e. *j*_*imp *_= *j*_*exp *_= *j *and *j*(*t*) is constant in time. Also, during the short period of photobleaching *j *remains constant because the bleaching process only destroys the fluorescing dye but not the molecule of interest. By combining equation (8) with the equilibrium condition and the ansatz c_cyt/nuc,L_(t) = c_cyt/nuc,0_(t)e^-εt^, equation (16) transforms into

(17) $$\left(\begin{array}{c} c_{cyt,0}\\ c_{nuc,0} \end{array}\right)=K\underset{=:M}{\underset{︸}{\left(\begin{array}{cc} -\frac{\alpha_{imp}}{V_{cyt}} & \frac{\alpha_{\exp}}{V_{cyt}}\\ \frac{\alpha_{imp}}{V_{nuc}} & -\frac{\alpha_{exp}}{V_{nuc}} \end{array}\right)}}\left(\begin{array}{c} c_{cyt,0}\\ c_{nuc,0} \end{array}\right)$$

with $\alpha_{imp}=\frac{j}{c_{cyt}}$ and $\alpha_{exp}=\frac{j}{c_{nuc}}$. This linear ODE can be solved. The system has a constant solution

(18) $$V_{cyt}c_{cyt,0}(t)+V_{nuc}c_{nuc}{}_{,0}(t)\;=N_{tot}=const.$$

corresponding to the eigenvalue λ_1 _= 0 of *M *and a solution

(19) $$\left(\begin{array}{c} c_{cyt,0}\\ c_{nuc,0} \end{array}\right)(t)\propto \left(\begin{array}{c} V_{cyt}\\ -V_{nuc} \end{array}\right)e^{-\lambda_{2}t}$$

with $\lambda_{2}=\frac{\alpha_{imp}}{V_{cyt}}+\frac{\alpha_{exp}}{V_{nuc}}$.

In the experiment, fluorescent signals *S*_*cyt *_and *S*_*nuc *_are measured. The signal

(20) $$S=f(t)c_{L}(t)=f(t)e^{-\epsilon t}c_{0}(t)$$

depends linearly on the concentration; the scaling factor *f *between concentration and signal may be time dependent. In order to get rid of the scaling factor and ε, new variables *cyt *and *nuc *are introduced and transformed using eq. (20):

(21) $$cyt:=\frac{V_{cyt}S_{cyt}}{V_{cyt}S_{cyt}+V_{nuc}S_{nuc}}=\frac{V_{cyt^{c}cyt,0}}{N_{tot}}$$

(22) $$nuc:=\frac{V_{nuc}S_{nuc}}{V_{cyt}S_{cyt}+V_{nuc}S_{nuc}}=\frac{V_{nuc^{c}nuc,0}}{N_{tot}}.$$

Consequently, the experimentally accessible quantities *cyt *and *nuc *are directly associated to the concentrations appearing in the ODE system. Note that the exponential decrease of the signal (due to continuous bleaching) and the proportionality factor between the signal *S *and the concentration *c*_*L *_drop out. This is even true if the proportionality factor is time dependent.

Using both eq. (19) and the experimental quantities *cyt *and *nuc*

(23) $$cyt(t)\cdot \frac{V_{nuc}}{V_{cyt}}-nuc(t)\cdot \frac{V_{cyt}}{V_{nuc}}=a_{0}e^{-a_{1}t}+a_{2}$$

can be fitted to an exponential curve and the fit parameter *a*_1 _gives the desired result

(24) $$\alpha_{imp}=\frac{a_{1}}{K\left(\frac{1}{V_{cyt}}+\frac{c_{cyt}}{c_{nuc}}\frac{1}{V_{nuc}}\right)}$$

(25) $$\alpha_{exp}=\frac{a_{1}}{K\left(\frac{1}{V_{nuc}}+\frac{c_{nuc}}{c_{cyt}}\frac{1}{V_{cyt}}\right)}.$$

The resulting rate function *α*_*l*_(*c*) or equivalently *j*_*l*_(*c*) = *c*α_*l*_(*c*) can be used for microscopy data: cell images are analyzed for the quantity *K *and for fluorescence intensities which allow calculating the protein concentrations of interest. Plugging these values in the formula for *j*_*l*_(*c*) yields an estimate for the current between nucleus and cytoplasm of the investigated cell without measuring it explicitly.

## Authors' contributions

ACP and DK conceived and designed the methodology. ACP generated all biological data and wrote parts of the manuscript. DK devised the mathematical model, developed the software and wrote parts of the manuscript. JB established the Tet-inducible expression system. UK and JT participated in the design of the methodology and critiqued the manuscript. All authors read and approved the final manuscript.

## Supplementary Material

Additional file 1 **The file is an R script file designed for calibrating flow cytometry data to microscopy data**. A documentation of how to use the script is included in the header of the file. Click here for file

Additional file 2 **Sample config file for populist.R**. In the file "Purpose" can either be "Calibration" or "Measurement". "Method" is either "FACS" or "Microscopy". "Dox" refers to the preparation and can have arbitrary numbers. For every value of "Dox", an extra calibration is performed. "Intensity" and "Volume" refer to the column names of microscopy and flow cytometry data where intensity and volume values can be found. Finally, "File" is the filename of the data file that should be used. Click here for file
